# Supplementary material for: Impact of Environmental Conditions on the Protein Content of Staphylococcus aureus and Its Derived Extracellular Vesicles
Source: Microorganisms. 2022 Sep 9;10(9):1808. doi: 10.3390/microorganisms10091808 (PMC9506334; doi:10.3390/microorganisms10091808)
Supplement: Supplementary file 1 [file microorganisms-10-01808-s001.zip › Supplementary_Figures_Luz_microorganims.pdf]

Supplementary materials

Luz *et al*., Impact of environmental conditions on the protein content of *Staphylococcus aureus* and its derived extracellular vesicles

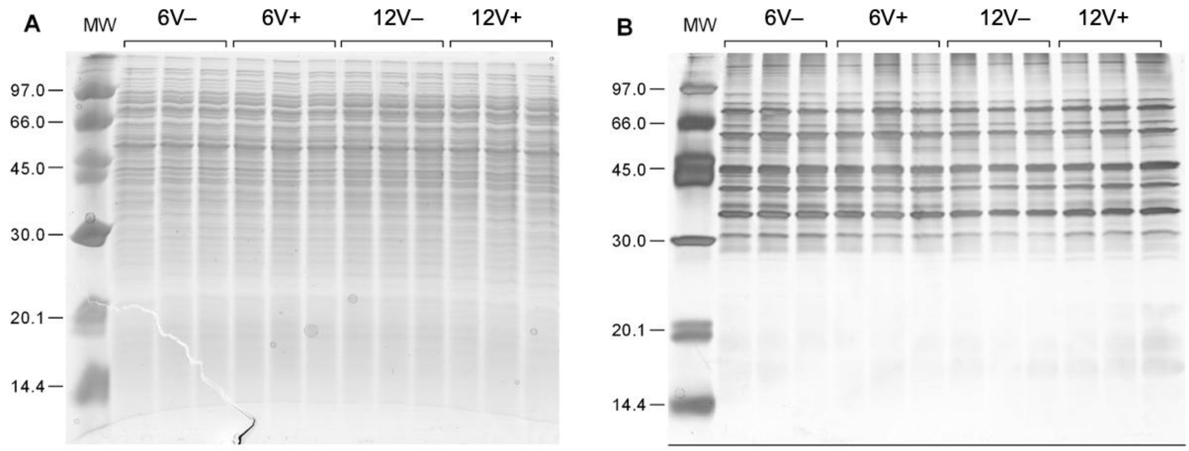

Figure S1. Protein profile of *S. aureus* HG003 WC and EVs. SDS-PAGE (12% gel) showing the protein profile of triplicates of WC (**A**) and EVs (**B**) in each condition. Molecular weight (MW) standards are indicated in kDa. Early- and late-stationary growth phases (6 and 12 h, respectively) in the absence (V−) or presence (V+) of vancomycin.

Supplementary materials

Luz *et al*., Impact of environmental conditions on the protein content of *Staphylococcus aureus* and its derived extracellular vesicles

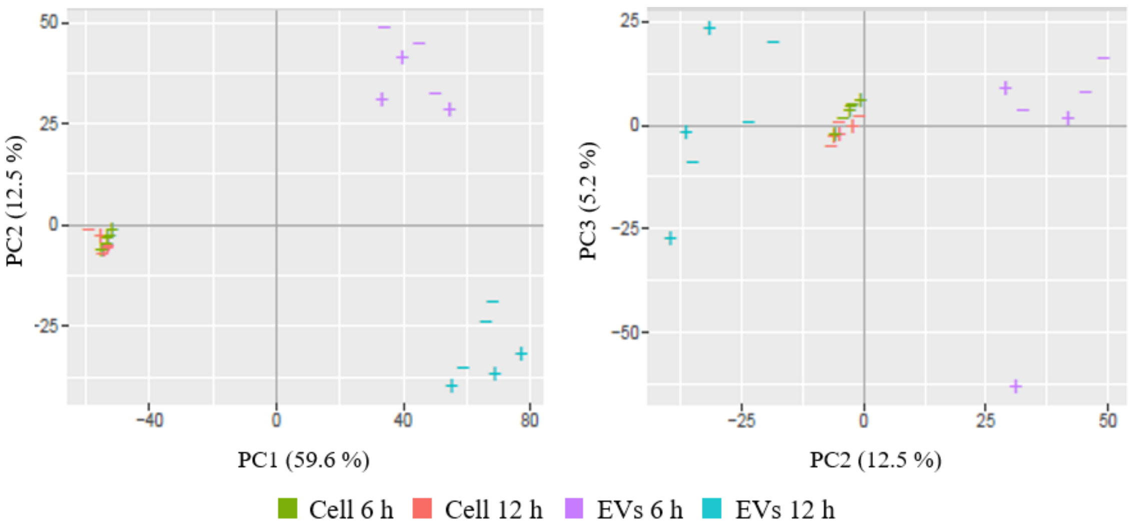

Figure S2. Principal component analysis (PCA) of peptide abundance in samples under each condition and across biological replicates. The percentages of variance that is associated with each axis are displayed. − and + corresponds to conditions in the absence and presence of vancomycin, respectively.

Supplementary materials

Luz *et al.*, Impact of environmental conditions on the protein content of *Staphylococcus aureus* and its derived extracellular vesicles

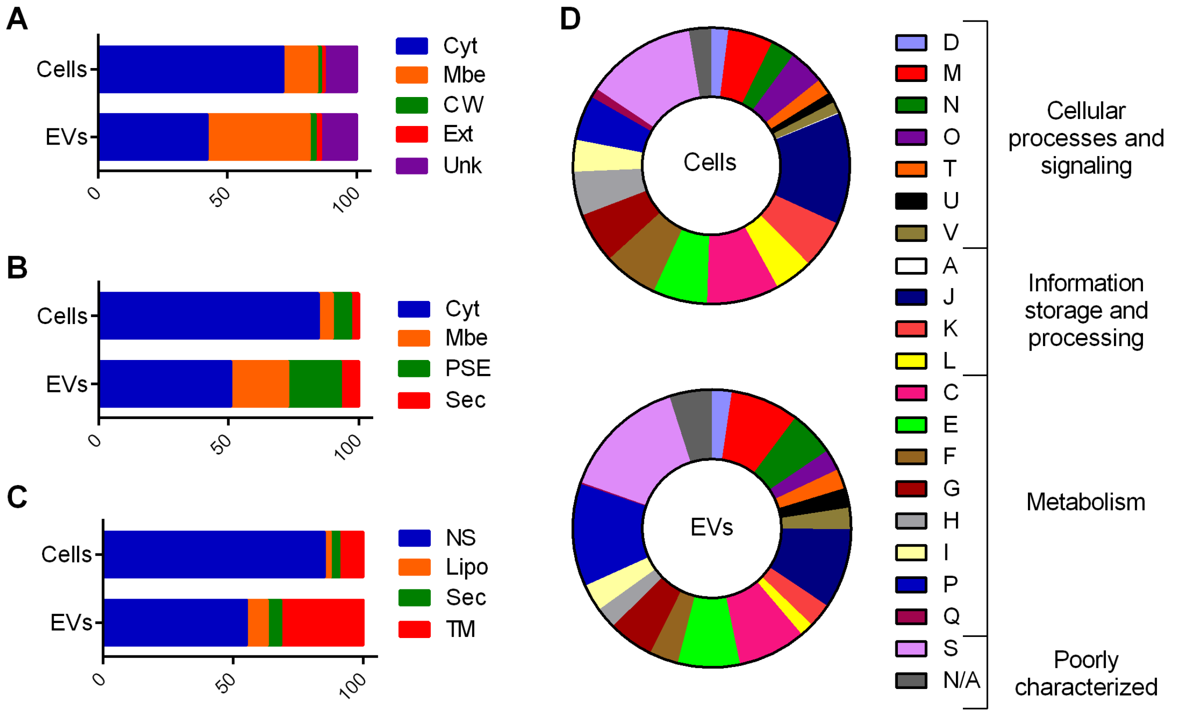

Figure S3. Comparative proteome analysis of *S. aureus* HG003 WC and EVs at 6 h without vancomycin (6V−). Prediction of sub-cellular locations of proteins with PSORTb (**A**), SurfG+ (**B**), and PRED-LIPO (**C**), and the prediction of COG functional categories of proteins with eggNOG-mapper (**D**). Cyt, cytoplasmatic; Mbe, membrane; CW, cell wall; Ext, extracellular; Unk, unknown; PSE, surface-exposed; Sec, secreted; NS, no signals found; Lipo, lipoprotein; TM, transmembrane; COG functional categories: D, Cell cycle control, cell division, chromosome partitioning; M, cell wall/membrane/envelope biogenesis; N, cell motility; O, Post-translational modification, protein turnover and chaperones; T, signal transduction mechanisms; U, Intracellular trafficking, secretion and vesicular transport; V, defense mechanisms; A, RNA processing and modification; J, translation, ribosomal structure and biogenesis; K, transcription; L, replication, recombination, and repair; C, energy production and conversion; E, amino acid transport and metabolism; F, nucleotide transport and metabolism; G, carbohydrate transport and metabolism; H, coenzyme transport and metabolism; I, lipid transport and metabolism; P, inorganic ion transport and metabolism; Q, secondary metabolites biosynthesis, transport, and catabolism; S, function unknown; NA, not available

Supplementary materials

Luz *et al.*, Impact of environmental conditions on the protein content of *Staphylococcus aureus* and its derived extracellular vesicles

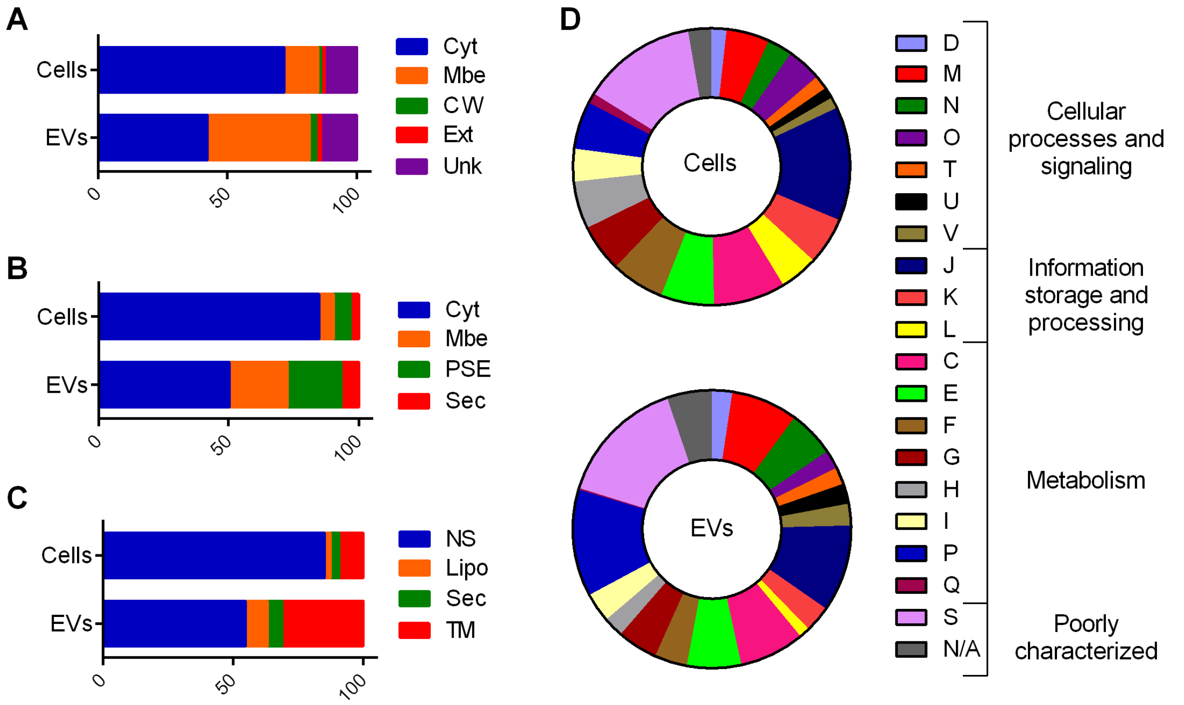

Figure S4. Comparative proteome analysis of *S. aureus* HG003 WC and EVs at 6 h with vancomycin (6V+). Prediction of subcellular locations of proteins with PSORTb (**A**), SurfG+ (**B**), and PRED-LIPO (**C**), and prediction of COG functional categories of proteins with eggNOG-mapper (**D**). For legend, please refer to Figure S3.

Supplementary materials

Luz *et al*., Impact of environmental conditions on the protein content of *Staphylococcus aureus* and its derived extracellular vesicles

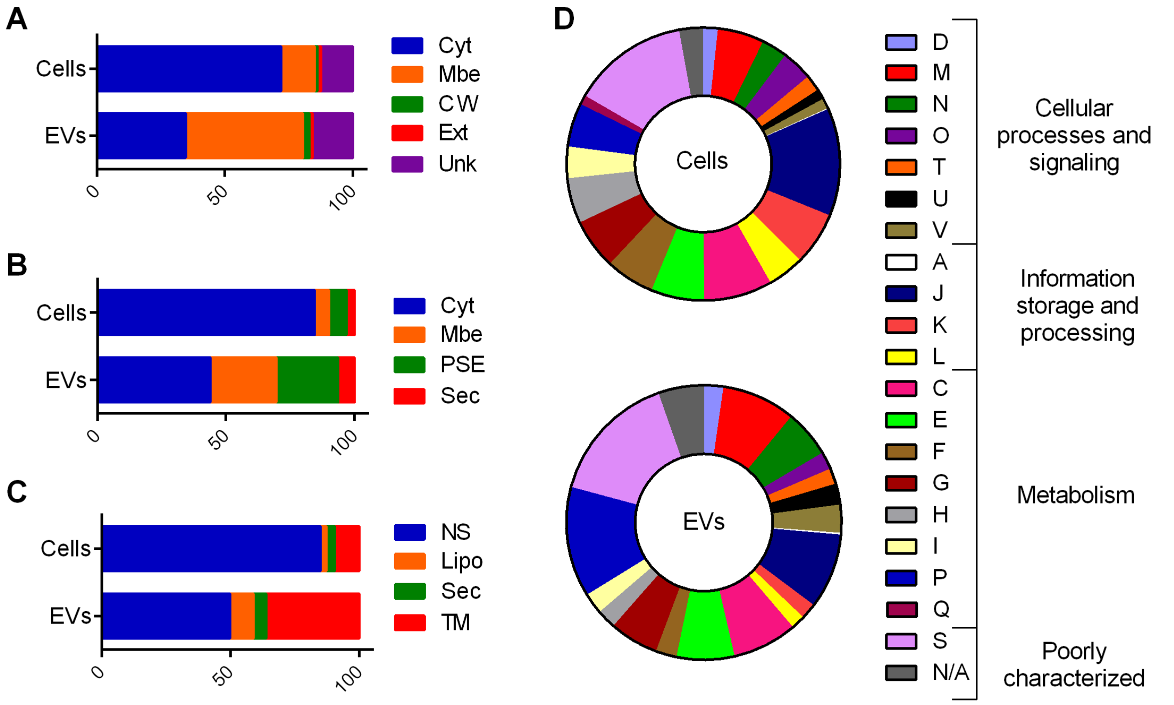

Figure S5. Comparative proteome analysis of *S. aureus* HG003 WC and EVs at 12 h without vancomycin (12V−). Prediction of subcellular locations of proteins with PSORTb (**A**), SurfG+ (**B**), and PRED-LIPO (**C**), and prediction of COG functional categories of proteins with eggNOG-mapper (**D**). For legend, please refer to Figure S3.

Supplementary materials

Luz *et al*., Impact of environmental conditions on the protein content of *Staphylococcus aureus* and its derived extracellular vesicles

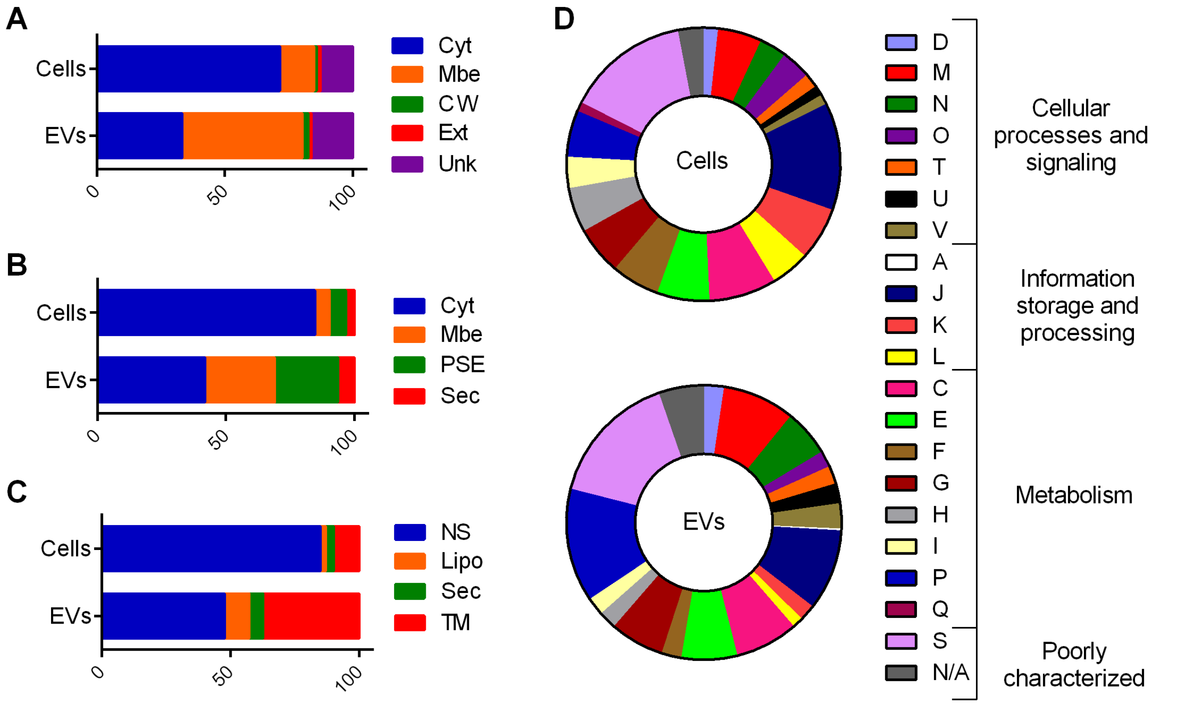

Figure S6. Comparative proteome analysis of *S. aureus* HG003 WC and EVs at 12 h with vancomycin (12V+). Prediction of subcellular locations of proteins with PSORTb (**A**), SurfG+ (**B**), and PRED-LIPO (**C**), and prediction of COG functional categories of proteins with eggNOG-mapper (**D**). For legend, please refer to Figure S3.
